# Ultrasound-Guided Regional Block in Renal Transplantation: Towards Personalized Pain Management

**DOI:** 10.3390/jpm15090411

**Published:** 2025-09-02

**Authors:** Ahmad Mirza, Munazza Khan, Zachary Massey, Usman Baig, Imran Gani, Shameem Beigh

**Affiliations:** 1 Department of Transplant Surgery, Wellstar MCG Health, Augusta, GA 30912, USA; zmassey@augusta.edu; 2 Medical University–Pleven, 1, Saint Kliment Ohridski Street, 5800 Pleven, Bulgaria; 3 Department of Nephrology, Hypertension and Transplant Medicine, Wellstar MCG Health, Augusta, GA 30912, USAsbeigh@augusta.edu (S.B.)

**Keywords:** ultrasound, anesthesia, regional block, kidney transplantation, perioperative pain, postoperative analgesia, nerve block, pain control

## Abstract

**Introduction:** The management of peri-operative pain significantly impacts the post-operative recovery following kidney transplant. For decades, regional blocks have been utilized for post-operative pain management following abdominal surgery. The data on the routine use of regional blocks peri-operatively during kidney transplants are limited. We aim to review our current clinical practice of peri-operative use of regional blocks during kidney transplants and management of peri-operative pain up to 24 h. **Methods:** A consecutive series of 100 patients who underwent kidney transplant was reviewed. All demographic data including patient’s age, gender, race, and body mass index were collected. Pre-transplant co-morbidities were summarized for all patients and included the American Society of Anesthesiologists (ASA) score. Patients were divided into two groups based on whether they received a transversus abdominis plane (TAP) block. Group A consisted of patients who received an ultrasound-guided TAP block, while Group B included patients who did not receive any form of TAP block. The intra-operative and post-operative use of analgesia was recorded for up to 24 h post kidney transplant. All peri-operative complications were reviewed. The chi-square test and Fisher’s exact test was used to compare symptoms (nausea, vomiting, and pruritus) between the two groups. Similarly, the use of analgesia was also compared. **Results:** A total of 100 patients were identified and equally distributed between the two groups [Group A = 50 (TAP block), Group B = 50 (non-TAP block)]. There was a statistically significant reduction in the use of intraoperative fentanyl (*p* = 0.04) in Group A. There was no difference in the post-operative use of hydromorphone (*p* = 0.665), oxycodone (*p* = 0.75), and acetaminophen (*p* = 0.64) up to 24 h after the kidney transplant procedure. There was no difference between post-operative nausea (*p* = 0.766), vomiting (*p* = 0.436), and pruritus. There were no complications recorded secondary to the use of regional blocks in Group A. **Conclusions:** The use of regional anesthesia in kidney transplant recipients is a safe approach without complications. The study concluded that regional blocks decrease the use of intra-operative opioids. However, there was no difference in the use of post-operative requirements for analgesia or side effects up to 24 h after kidney transplant.

## 1. Introduction

The post-operative recovery can be enhanced by the management of peri-operative pain [1]. Goal-directed analgesia management post-surgery has become standard of care in enhancing post-operative recovery [1,2]. Various modalities of analgesics have been used to support early mobilization and reduce hospital stay. These approaches include analgesics administered orally, intravenously, or in fascial planes of surgery to decrease the overall analgesics requirements in the post-operative recovery phase [2]. Regional blocks have been used extensively in abdominal, orthopedics, and vascular surgery to provide immediate post-operative pain relief. The objective of regional blocks is to deliver local anesthetic (LA) in the immediate vicinity of operative field to block the nerve fibers transmission of pain signals. The effect of regional blocks can last up to 24 to 72 h, depending on the agent used and the concentration of LA administered. However, the data for use of regional blocks in kidney transplant recipients is limited. No formal consensus or guidelines have been reached for peri-operative patient management, with significant institutional variation [2]. Kidney function post-transplant is slow to recover, with a significant proportion of patients still requiring dialysis. Non-steroidal anti-inflammatory drugs are avoided after kidney transplant due to their potential nephrotoxicity, as they exacerbate acute kidney injury (AKI) [3,4]. Opioid-based analgesia delivered via patient-controlled analgesic (PCA) regimes are the most widely practiced method for pain management [5]. However, PCAs are also associated with significant side effects related to opioid sue. The use of opioids immediately post-operatively is also associated with the incidence of nausea, vomiting, dependence, decreased gut mobility, and respiratory depression [3,4,5]. In kidney transplant recipients, opioids can modify renal blood flow and sodium clearance, while also causing extra-renal side effects, including constipation, pruritus, nausea, and vomiting [6,7].

The use of ultrasound-guided regional nerve blocks has become increasingly common approach for peri-operative pain control. The use of a regional block (transverse abdominis block—TAP) has been described in many abdominal surgeries, including colectomies, cholecystectomies, hysterectomies, and hernia repair. TAP blocks exert their effect on the T6-L1 thoracolumbar nerves, providing anesthesia to the anterolateral abdominal wall [8]. TAP blocks as well as erector spinae and quadratus lumborum blocks have been employed to provide intraoperative anesthesia in kidney transplant surgeries [9,10,11,12]. When the LA agents are delivered to correct fascial plane, their administration can help to improve immediate post-operative analgesic requirements. Their use has been employed for reduction in the use of intra- and post-operative analgesics. Data on the use of regional blocks in kidney transplant patients is mixed with some studies suggesting clear benefit and others showing no clinical significance [13].

This retrospective study aims to review our current clinical practice of the use of regional blocks in kidney transplant recipients. The study also aims to assess the use of intra-operative and up to 24-h post-operative analgesics in kidney transplant patients with and without regional block. The study also aims to explore the possible complications and side effects related to the use of a TAP block in kidney transplant recipients. The study also aims to explore need for future studies to identify the benefits of transplant patients in a larger cohort of patients. The findings will contribute to the growing body of data on perioperative pain management in the patient population requiring renal transplantation and support the development of a more personalized approach for this specific patient group.

## 2. Methods

The study was conducted at Wellstar MCG, Augusta University, Augusta, Georgia, USA. Full research authorization was obtained from the research review board. These retrospective data were collected form a consecutive series of 100 patients who underwent kidney transplant. No findings altered patient’s standard management since all patients have already completed their standard treatment. All patients underwent a deceased donor kidney transplant. All patients were listed on United Network of Organ Sharing (UNOS, 700 N 4th St. Richmond, VA 23219, USA) national list and were offered transplant from the national organ matching scheme. Adult patients with ages of ≥18 were included in the study. Patients with opioid dependence were excluded from the study. Opioid dependence was regarded as a confounding variable; therefore, all patients with a history of opioid dependence and usage were excluded.

All demographic data including patient age, gender, race, and body mass index were collected. Pre-transplant co-morbidities were summarized for all patients. All patients charts were extensively reviewed to obtain all information pertinent to the research. Based on the data obtained, patients were divided into two groups, namely, the regional block versus non-block group. For both groups, the intra-operative and post-operative use of analgesia was recorded for up to 24 h post kidney transplant. All peri-operative complications were reviewed for all patients and were included in the analysis.

### 2.1. Data Analysis

All data were collected using a standardized excel sheet with the data stored at the central human resource box drive of Augusta University. The data were verified for accuracy by two separate observers. IBM SPSS Statistics version 29 (IBM, New York, NY, USA) software was used to compare both continuous and discrete data variables.

Normality of the data was assessed, and either a parametric (*t*-test) or non-parametric (Mann–Whitney U) test was applied. Fisher’s exact tests and chi-square tests for a standard table comparison were used to compare symptoms (nausea, vomiting, and pruritus) between the two groups. A *p* value of <0.05 was regarded as significant.

### 2.2. Procedure of Regional Block

Informed consent for the procedure was completed before the administration of regional blocks. All patients were explained the procedure, complications, and expected outcomes. It was important to make patients aware that they will receive additional analgesia as per the clinical need along with the regional block. All patients were also made aware that the expected outcomes for pain improvement may not be achievable with the regional blocks. All patients were also given an opportunity to opt out of regional block administration.

**Figure 1:**
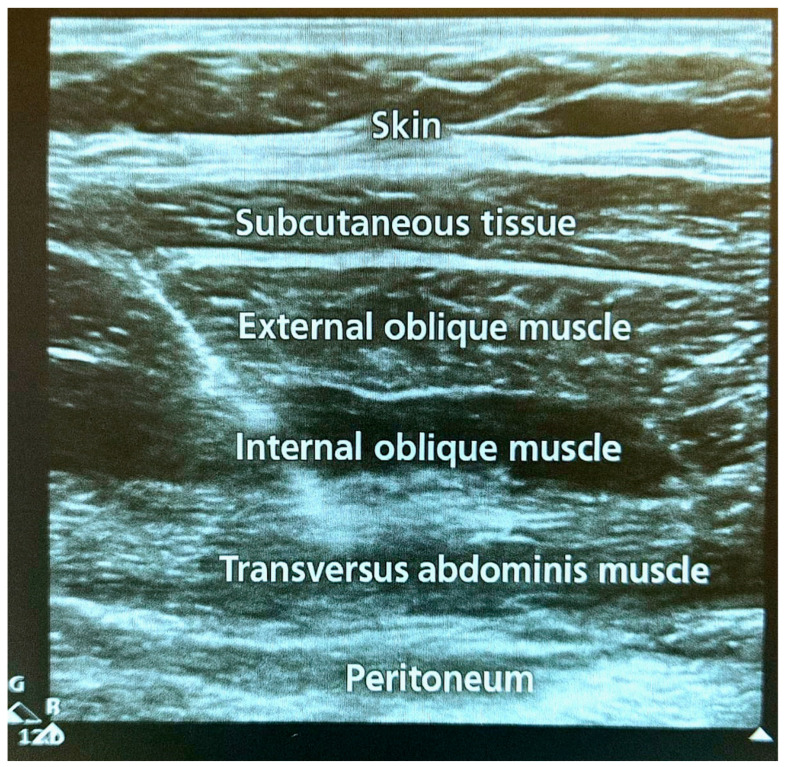
Ultrasound-guided insertion of the needle for transversus abdominis plane block to deliver local anesthesia.

All procedures were either performed or supervised by a consultant anesthetist. A total of 4 medical personnel (consultant, intern, assistant, and nurse practitioner) were present during the procedure. For a standard regional block, the patient was positioned supine on the operating table, and the abdomen was exposed. Sterile preparation of the site was performed in the usual fashion with gowns, gloves, and masks. The chlorhexidine scrub was used to clean the site. The ultrasound probe (Philips SPARQ Ultrasound, L12-4 Probe, Cambridge, MA, USA) was placed in a transverse plane at the midaxillary line on the right side and was used to identify the muscle layers (external oblique, internal oblique, and transversus abdominis). A 20-gauge sterile needle was inserted under ultrasound guidance into the transversus abdominal plane between the internal oblique and transversus abdominis muscles (Figure 1). Once the needle was in the correct plane, aspiration was performed to ensure there was no blood or fluid return. Approximately 5 mL of 0.5% ropivacaine was injected, and spreading was visualized under ultrasound in the plane to confirm the correct location. Once the location was confirmed, the remaining 15 mL of 0.5% ropivacaine was injected. The procedure was then repeated on the left side.

A sterile dressing was placed at the site of the insertion of the needle, which was subsequently removed. Immediate attention was paid for any evidence of swelling, bruising, hematoma, or cardiac or neurological symptoms related to LA administration and toxicity. In addition, any evidence of local skin irritability and dermatological reaction at the site of administration of LA was also monitored. All patients were observed in a monitored post-surgery recovery area up to four hours after completion of kidney transplant. Patients still had invasive monitoring in the form of an arterial line and two large intravenous accesses in situ. Patient’s vitals were monitored with both invasive (arterial line) and non-invasive approaches with a blood pressure cuff.

## 3. Results

A total of 100 patients were identified and were equally distributed between the two groups [(Group A = 50 (block), Group B = 50 (no block)]. The mean calculated age range for all patients in the study was 53.4 years (range 26 to 75 years). There was no difference between the mean age (*p* = 0.85) of patients in Group A (27 to 73 years) when compared to Group B (26 to 75 years) (Table 1). The mean BMI for all patients in the study was 29.4 kg/m^2^ (range 17 to 40 kg/m^2^). There was no difference in the BMI (*p* = 0.77) between the patients in Group A (mean = 29.27 kg/m^2^, range 17 to 39 kg/m^2^) and Group B (mean = 29.58 kg/m^2^, range 17 to 40 kg/m^2^). On further sub-group analysis, there was no difference identified in the gender distribution, race, co-morbidities, and American Society of Anesthesiologists (ASA) score between the two groups.

There was a noticeable reduction in the use of intraoperative fentanyl (*p* = 0.04) in Group A with pre-operative regional block (Table 2). However, the analysis failed to show a difference in the post-operative use of hydromorphone (*p* = 0.66), oxycodone (*p* = 0.75), and acetaminophen (*p* = 0.64). At 24 h, there were no complications recorded secondary to the use of regional blocks in Group A. In addition,, there was no difference between post-operative nausea (*p* = 0.766), vomiting (*p* = 0.436), and pruritus (Table 3).

All patients at the 4-week clinic follow-up after deceased donor kidney transplant had no complaint of numbness, motor weakness, or persistent paresthesia. However, seven patients (Group A = 4, Group B = 3) reported numbness at the incision site. However, none of these complaints were related to the use of regional block. The complaint of numbness at the site of incision is a common finding and is related to scar tissue formation and regeneration. The numbness usually resolves after a period of few months with both tissue and nerve regeneration.

There were no reported episodes of hematoma, bruising, or tingling at the site of LA administration. There were no anaphylactic episodes related to the administration of LA. There were no episodes of systemic toxicity related to LA usage. All patients tolerated the procedure of administration of LA well. Most patients complained of initial pain at the site of insertion of needle through the skin. However, no further pain episode was noted once the needle is in the facial plane. The mean time of completing the procedure was 16 min (range 12 to 21 min).

## 4. Discussion

Post-operative pain can have a significant impact on post kidney transplant recovery. For management of pain, transplants patients are offered a combination of oral, intravenous, and regional blocks. Regional blocks have been used in conjunction with other analgesics to achieve adequate pain control in recipients of kidney transplants [13]. In kidney transplant patients, the literature has shown faster recovery times with corresponding cost-savings in patients who received regional blocks. However, there is considerable variation in outcomes and reproducibility of results from use of LA for regional blocks. An international study of 286 kidney transplant patients comparing the clinical benefit of regional block identified a significant reduction in length of hospital stay for both live donor and deceased donor kidney transplants [13]. Similar findings were also reported by Ali et al. [14].

Regional blocks in kidney transplant patients have been associated with reduction in number of inpatient stays and therefore result in considerable cost-savings. The study analyzed a group of 46 kidney transplant recipients who were administered ultrasound-guided TAP blocks. The TAP block patient group had on average of 2 less days of hospital stay compared to a matched control group. There were no reported complications from the use of TAP block in kidney transplant recipients. The study also demonstrated significant cost-savings [14]. However, more research needs to be conducted to confirm the cost effectiveness of use of regional block in kidney transplant patients.

The efficacy of regional blocks in reducing post-operative pain and opioid consumption has also been studied. A systematic review and meta-analysis evaluated 51 randomized control trials (RCTs) of abdominal surgeries that employed TAP block versus control analgesics [15]. The study has shown that TAP blocks were associated with reduced pain scores at 6, 12, and 24 h. The study also showed a significant decrease in 24-h morphine consumption. However, some individual RCTs in the same review also showed certain opioids as having greater analgesic effects than the TAP block, making the findings of this meta-analysis non-unanimous [15]. This leads to the idea of further research and studies to incorporate kidney transplant patients undergoing regional blocks for peri-operative pain management. This approach will help to improve outcomes and peri-operative pain control in patients undergoing kidney transplant.

Adding epinephrine to local anesthetics in TAP blocks slows systemic absorption, lowering peak plasma concentrations and delaying time to peak, which may reduce the risk of toxicity [16,17]. In renal transplant patients, a study showed a significant reduction in opioid requirements with bupivacaine plus epinephrine compared to control [18]. No complications were reported in this group as well. While generally safe and potentially beneficial, more data on the renal transplant population are still needed [19,20]. In our cohort of patients, no epinephrine was included with the LA agent. However, a future study incorporating a randomized controlled trial in kidney transplant patients can also focus on the use of epinephrine versus no epinephrine usage with LA for the administration of regional blocks.

It is also documented that opioid use during kidney transplant surgeries has been associated with an increased risk of graft failure and mortality [21]. Morphine’s active metabolite morphine-6-glucuronide (M6G) and fentanyl can accumulate in the body due to reduced clearance post kidney transplant, which may lead to significant side effects, i.e., ventilatory depression, dependence, and addiction [22]. Therefore, there can be a significant future role in the use of regional blocks routinely during kidney transplant for the purpose to decrease peri-operative morbidity associated with the use of opioid-based analgesia.

One of the outcomes measured in our study was the use of intra-operative fentanyl. Our study identified significantly reduced intra-operative fentanyl use in patients who received (Group A) compared to patients who did not receive the block (Group B). This finding is in line with other abdominal surgery studies, where regional blocks have been shown to decrease intra-operative opioid requirements in cholecystectomies, hepatectomies, and pancreaticoduodenectomies [23,24,25]. In kidney transplantations, studies by Mohammadi et al. and Mukhtar et al. have shown a reduction in the intra-operative fentanyl (*p* = 0.001) and morphine (*p* < 0.0001) requirements in patients receiving TAP blocks, respectively [18,26]. The decrease in intra-operative opioid use seen after administering TAP blocks supports its consideration for routine use in kidney transplant surgeries.

Complications associated with TAP blocks are uncommon. Those that have been reported include bowel perforation, infection, intravascular injection, inadvertent visceral injection, and femoral nerve palsy. With ultrasound guidance now standard of practice, the incidence of these complications has markedly decreased. During the review of our analysis, none of the immediate or short-term (up to 4 weeks) complications were identified to be related to the use of regional blocks. Our results align with existing evidence, supporting the continued use of TAP blocks as a safe and effective technique [20,27].

The role of regional blocks in reducing first 24-h post-surgery opioid consumption and opioid-related side-effects is lacking. A systematic review and meta-analysis of 17 papers showed that TAP block groups had reduced visual analog scale (VAS) pain scores and opioid consumption 24-h post kidney transplant [9]. Opioid prescription at discharge is also observed to be reduced in patients who received TAP blocks [21]. However, some studies have also shown the effectiveness of the TAP block to reduce over time. Yang et al. reported the use of intravenous morphine in PCA with a morphine PCA plus TAP block [13]. The study found no significant difference in overall morphine consumption post kidney transplant between both groups at 24 h [13]. Another study showed that VAS pain scores recorded at 24 h in both static and dynamic positions had no statistical difference between the standard anesthesia and TAP block group [28]. However, a more recent literature analysis is required to identify the current association and significant benefit achieved with the use of regional blocks in kidney transplant patients.

Continuous TAP blocks have also been successfully used to provide analgesia post kidney transplant. Inserted intra-operatively or through ultrasound guidance, continuous TAP blocks have reduced pain scores and post-operative opioid consumption up to 48 h after surgery [29,30,31]. Further research is warranted to determine whether single-injection TAP blocks or continuous TAP blocks are superior in decreasing post-operative opioid use and pain intensity. This can be an area of potential future research, and the possibility of variability in the use of concentration and duration of LA in the continuous TAP block group should be explored. This future study can also focus on the duration of continuous LA administration from a TAP block.

In addition, our study did not find a statistically significant difference in the consumption of post-operative analgesics: hydromorphone, oxycodone, and acetaminophen. Similar results have also been described in a few RCTs. A study reported on 65 post kidney transplant patients who were either randomized to receive either a TAP block or a hoax block of 0.9% saline. Morphine utilization measured at 24 h did not statistically differ between the two groups (*p* = 0.817) [32]. Likewise, a RCT on 51 post kidney transplant patients found that patients who received a TAP block had similar median morphine requirements compared to the control group (19.4 mg vs. 16.4 mg, *p* = 0.94), showing no conferring benefit related to the addition of a TAP block [33]. Our study also recorded the incidence of nausea, vomiting, and pruritus in both the regional block (Group A) and non-block (Group B) patients. The incidence of these side effects did not significantly differ between the two groups, possibly due to both groups having similar post-operative analgesic consumption. In studies with a statistically significant difference in post-operative opioid consumption between regional block groups and control groups, the frequency of side effects varied. Either the control group reported a greater incidence of side effects or no difference between the two groups was reported [9,13,29].

Various local anesthetic agents (LAs)are used for TAP blocks. The mechanism of action of LAs include inhibition of the influx of sodium ions through sodium channels in nerve fibers. This inhibition blocks nerve transmission by preventing the nerve from depolarizing and initiating an action potential. The degree and duration of nerve blockade depends on multiple factors, e.g., the type of LA used, concentration, etc. A complete evaluation of the patient’s medical history, most importantly cardiac and anti-coagulation history, is crucial in the assessment, management, and administration of regional blocks. Precise and proper administration of local anesthetic agent under ultrasound guidance helps to guide correct needle placement targeting nerve fibers in the exact fascial layer to achieve maximum blockage. Post administration monitoring is crucial to identify early complications and immediate steps taken to prevent worsening [34]. If all the above steps are taken into consideration, this forms the basis of a personalized approach and practice.

This study has several significant limitations, including its single-center design, which limits generalizability to other institutions and patient populations. Its retrospective nature relies on potentially incomplete or inconsistent chart documentation and introduces inherent data collection bias. The lack of randomization in group allocation creates selection bias and fails to control for confounding variables that could influence the outcomes between the regional block and non-block groups.

Future prospective, multicenter randomized controlled trials with larger sample sizes are needed to provide more evidence regarding the efficacy of regional blocks in kidney transplant patients. In addition, a future study should be conducted also comparing different types of regional blocks and different anesthetic agents. The comparison can help to identify the usefulness and duration of blocks offered by different anesthetic agents. This future study should also compare the role of continuous block with local anesthetic agent infiltration versus a single-block approach, which has been the practice at our facility.

## 5. Conclusions

Pain management is an important consideration when facilitating post kidney transplant recovery. Regional blocks have been previously described to lower pain scores and opioid requirements in peri-operative kidney transplant patients. Our study found that the addition of a single-injection TAP block to our analgesic regime decreased intra-operative opioid use but did not reduce 24-h post-operative analgesic consumption. The incidence of post-operative nausea, vomiting, and pruritus also remained similar in both the regional blocked and non-blocked group. It is important to note that our current study is also recommending a future randomized control trial comparison of the single-block approach versus continuous infiltration with local anesthetic of the incision via deep facial catheters for a period of up to 72 h after kidney transplant. This approach may help to decrease the post-operative analgesic requirements for a longer duration and form the basis of personalized medicine individually modified to patients’ needs.

## Figures and Tables

**Table 1:** General demographic characteristics of the study population.

	Group A (Regional Block) n = 50	Group B (No Regional Block) n = 50
**Age** (years)	53.33 ± 12.53	53.48 ± 12.74
**Body mass index** (kg/m^2^)	29.27 ± 5.73	29.58 ± 5.10
**Gender**		
Male	31 (62.0%)	34 (68.0%)
Female	19 (38.0%)	16 (32.0%)
**Race**		
Black	36 (72.0%)	41 (82.0%)
Caucasian	11 (22.0%)	5 (10.0%)
Hispanic	2 (4.0%)	3 (6.0%)
Asian	1 (2.0%)	1 (2.0%)
**Cause of end-stage renal disease**		
Hypertension	18 (36.0%)	21 (42.0%)
Diabetes mellitus	17 (34.0%)	14 (28.0%)
Focal segmental glomerulosclerosis	1 (2.0%)	1 (2.0%)
Previous transplant	4 (8.0%)	3 (6.0%)
Autoimmune nephropathies	7 (14.0%)	9 (18.0%)
Polycystic kidney disease	3 (6.0%)	2 (4.0%)
**ASA score**		
3	40 (80.0%)	41 (82.0%)
4	10 (20.0%)	9 (18.0%)

**Table 2:** The use of analgesia perioperatively and up to 24 h after deceased donor kidney transplant. Total cohort values are presented for descriptive purposes only. Statistical comparison was performed between Group A and Group B.

Medication	All Subjects (Group A and B) n = 100	Group A (Regional Block) n = 50	Group B (No Regional Block) n = 50	*p*-Value (Group A vs. Group B)
Intraoperative fentanyl (μg)	186.75 ± 105.67	171.0 ± 93.32	202.5 ± 115.53	0.04
Intraoperative hydromorphone (mg)	0.72 ± 0.48	0.72 ± 0.49	0.71 ± 0.48	0.84
Hydromorphone (mg) in 24 h	0.97± 0.74	0.94 ± 0.80	1.007 ± 0.68	0.66
Oxycodone (mg) in 24 h	24.85 ± 16.07	25.6 ± 15.83	24.59 ± 16.22	0.75
Acetaminophen (grams) in 24 h	2.67 ± 0.89	2.71 ± 0.78	2.63 ± 0.99	0.64

**Table 3:** Occurrence of symptoms related to use of analgesia up to 24 h after deceased donor kidney transplant.

	Group A (Regional Block) n = 50	Group B (No Regional Block) n = 50	*p*-Value
**Nausea**			
Yes	7 (14.0%)	6 (12.0%)	0.766
No	43 (86.0%)	44 (88.0%)	
**Vomiting**			
Yes	5 (10.0%)	2 (4.0%)	0.436
No	45 (90.0%)	48 (96.0%)	
**Pruritus**			
Yes	2 (4.0%)	1 (98.0%)	1
No	48 (96.0%)	49 (2.0%)	

## Data Availability

The data that support the findings of this study are available on request from the corresponding author. The data are not publicly available due to privacy or ethical restrictions.

## Abbreviations

AKI	Acute kidney injury
ASA	American Society of Anesthesiologists
M6G	Morphine-6-glucuronide
PCA	Patient-controlled analgesia
RCT	Randomized controlled trial
TAP	Transversus abdominis plane
UNOS	United Network of Organ Sharing
VAS	Visual analog scale
